# Influences of State and Trait Affect on Behavior, Feedback-Related Negativity, and P3b in the Ultimatum Game

**DOI:** 10.1371/journal.pone.0146358

**Published:** 2016-01-07

**Authors:** Korbinian Riepl, Patrick Mussel, Roman Osinsky, Johannes Hewig

**Affiliations:** Department of Psychology 1, University of Würzburg, Würzburg, Germany; Middlesex University London, UNITED KINGDOM

## Abstract

The present study investigates how different emotions can alter social bargaining behavior. An important paradigm to study social bargaining is the Ultimatum Game. There, a proposer gets a pot of money and has to offer part of it to a responder. If the responder accepts, both players get the money as proposed by the proposer. If he rejects, none of the players gets anything. Rational choice models would predict that responders accept all offers above 0. However, evidence shows that responders typically reject a large proportion of all unfair offers. We analyzed participants’ behavior when they played the Ultimatum Game as responders and simultaneously collected electroencephalogram data in order to quantify the feedback-related negativity and P3b components. We induced state affect (momentarily emotions unrelated to the task) via short movie clips and measured trait affect (longer-lasting emotional dispositions) via questionnaires. State happiness led to increased acceptance rates of very unfair offers. Regarding neurophysiology, we found that unfair offers elicited larger feedback-related negativity amplitudes than fair offers. Additionally, an interaction of state and trait affect occurred: high trait negative affect (subsuming a variety of aversive mood states) led to increased feedback-related negativity amplitudes when participants were in an angry mood, but not if they currently experienced fear or happiness. We discuss that increased rumination might be responsible for this result, which might not occur, however, when people experience happiness or fear. Apart from that, we found that fair offers elicited larger P3b components than unfair offers, which might reflect increased pleasure in response to fair offers. Moreover, high trait negative affect was associated with decreased P3b amplitudes, potentially reflecting decreased motivation to engage in activities. We discuss implications of our results in the light of theories and research on depression and anxiety.

## Introduction

Social bargaining is important in our daily lives because we are reliant on receiving goods and services from others [1]. One important paradigm to study social bargaining behavior experimentally is the *Ultimatum Game* [2]. There, a proposer receives an amount of money and has to offer part of it to a responder. The proposer can either make unfair (e.g., offering 10% percent of the pot, keeping 90%) or fair offers (offering 50% of the pot). If the responder accepts the offer, both players receive the money as suggested by the proposer. If the responder rejects, none of the players gets anything.

Ultimatum Game research typically analyze the responders’ behavior [3]. Rational choice models [4] would predict that responders try to maximize their own financial gain. Therefore, they should accept any offer above zero because little gain is better than no gain. However, evidence indicates that people typically reject offers when they receive less than 30% of the pot [3], which seems irrational as participants reject donated money [5]. Possible explanations for this behavior are that participants typically experience anger when getting unfair offers [6], that accepting unfair offers might lower self-esteem [7], compared to rejecting them, and that participants may want to punish unfair proposers [8].

Psychologically informed studies have begun to investigate the role of emotions in decision making to arrive at a more comprehensive understanding of social bargaining. As such, previous studies suggest that state affect (i.e., momentarily emotions unrelated to the task itself) and trait affect (i.e., longer lasting affect) influence decisions in the Ultimatum Game, especially when responders receive unfair offers. When receiving fair offers, the acceptance rate is typically near 100%, regardless of affect.

Some studies indicate that negative state affect, compared to positive state affect, leads to decreased acceptance rates of unfair offers. Harlé and Sanfey [9] found that sadness, induced by short movie clips prior to the Ultimatum Game, led to lower acceptance rates, compared to neutral mood. Similar results were found for the comparisons between sadness and happiness [10], and anger compared to happiness [11]. Mussel, Göritz, and Hewig [12] used either a happy, a neutral, or an angry looking face of a proposer in each individual Ultimatum Game trial. They found higher acceptance rates in the happy and lower acceptance rates in the angry condition, compared to offers from neutrally looking proposers. Another study found higher acceptance rates of unfair offers after smiling, compared to non-smiling faces of proposers [13]. The face of the proposer may have served as an emotion inducing stimulus, although an alternative explanation would be that happy looking proposers were perceived as having more positive intentions and therefore their offers were more likely to be accepted [12].

Harlé and Sanfey [14], though, suggest that not the valence of emotions (i.e., positive vs. negative emotions) influence decisions in the Ultimatum Game, but rather their motivational direction. They found that approach-motivated state emotions (promoting engagement with offending stimuli; in their study amusement and anger) led to higher acceptance rates of unfair offers than withdrawal-motivated emotions (serenity and disgust). Valence, on the other hand, did not have any significant influence. Although this approach is consistent with some of the findings reported above and with a study showing that disgust decreases acceptance rates [15], it cannot explain why lower acceptance rates were found when participants were angry [11] or saw angry faces [12].

It seems to be relatively clear, however, that state happiness (a positive approach-motivated emotion) leads to higher acceptance rates, whereas state sadness (a negative withdrawal-motivated emotion) leads to lower acceptance rates of unfair offers. Interestingly, an exactly opposite pattern has been reported when it comes to trait affect, that is, longer-lasting emotional dispositions. Trait affect can be measured by the *Positive and Negative Affect Schedule* (PANAS) [16]. This questionnaire contains two distinct dimensions, positive affect and negative affect. Dunn, Makarova, Evans, and Clark [7] found that high trait positive affect was associated with lower acceptance rates to unfair offers, whereas elevated trait negative affect was associated with higher acceptance rates. Dunn et al. discuss that high self-esteem might explain these results in those who are high in trait positive affect, whereas those high in trait negative affect may think that they have to “take the crumbs from under the table” [7]. Harlé, Allen, and Sanfey [17] found that depressed individuals accepted more unfair offers than non-depressed people. Depression is linked to both decreased trait positive affect and increased trait negative affect [7, 18], which can consolidate the two findings mentioned above.

To sum up, diverging results were found for state and trait affect, indicating that different mechanisms are in play. However, effects of state and trait affect have thus far not been investigated simultaneously, including the investigation of potential interactions. In our study, we were interested in examining the state emotions happiness, anger, fear, and neutral. We hypothesized that state happiness (a positive approach-motivated emotion) would lead to increased acceptance rates, whereas state fear (a negative withdrawal-motivated emotion) would lead to decreased acceptance rates, compared to a neutral mood. As anger is both negative and approach-motivated, we had no clear hypothesis regarding its impact on acceptance rates, and investigated its effect exploratively. With regard to emotional dispositions, we hypothesized an opposite effect, namely that high trait positive affect would lead to decreased acceptance rates, whereas high trait negative affect would lead to increased acceptance rates. We hypothesized that all these effects should be especially true for unfair Ultimatum Game offers. Additionally, we investigated exploratively whether interactions between state and trait affect would occur.

The investigation of the neural correlates of social behavior has recently received considerable attention [19] as it provides an additional level of analysis and might ultimately provide explanations for the behavioral effects. Therefore, apart from examining behavior, we were interested in whether the differential influences of state and trait affect on acceptance behavior are reflected in corresponding neural processing. An interesting event-related potential in this context is the feedback-related negativity (FRN). The FRN occurs when participants receive negative feedback and presumably has the same source as the *error-related negativity* that occurs, among others, in choice reaction time tasks when participants detect by themselves that they had just made a wrong choice [20, 21]. Importantly, several studies have shown that unfair Ultimatum Game offers elicit larger FRN amplitudes compared to fair offers, probably because they represent more negative feedback on what the proposer is willing to offer to the responder [13, 22–26]. Mussel et al. [13] found smaller FRN amplitudes to unfair Ultimatum Game offers when proposers were smiling, compared to non-smiling. However, to our knowledge no study has investigated the influences of state affect, induced prior to the Ultimatum Game, and simultaneously trait affect on the FRN in the Ultimatum Game yet.

In addition, we were interested in investigating the P3b. This component originates from temporal-parietal regions and seems to be associated with attentional processes (e.g., it is larger when a stimulus is a target) and memory [27]. Moreover, it has been found to be sensitive to the magnitude of monetary reward in a card gambling task [28] and has been linked to motivational relevance of stimuli [29]. As fair offers are more psychologically rewarding than unfair offers [30], they might thus induce larger P3b amplitudes. In line with that, Ma et al. [26] found larger P3b amplitudes after fair, compared to unfair offers. Moreover, the experience of different state and trait emotions might alter motivational processing, resulting in changes in the P3b component.

## Methods

### Participants

Forty participants (26 female, 14 male) were recruited via announcement on a local website. Their age ranged from 19 to 34, with a mean age of 24.4 years (SD = 3.1 years). They had diverse educational backgrounds (four participants had a secondary school diploma, 26 had a university entrance degree, nine had a university degree and one had another educational attainment).

Participants were told that they would receive their earnings in the Ultimatum Game plus an extra compensation of 6 € in return for their participation. One undergraduate psychology student was additionally granted course credit. The whole experiment took between 2 and 2.5 hours. All participants gave written informed consent for participation. The study was approved by the ethics committee of the German Psychological Society.

### Procedure

After having filled out a questionnaire on socio-demographic data, participants were individually seated in a sound attenuated electroencephalogram (EEG) cabin. In order to set up a cover story, a photo of each participant was taken and they were asked to make 12 Ultimatum Game offers as proposers. As in the later game, they were only allowed to make 1, 3, or 5 Cent offers out of a 10 Cent pot. They were told that future participants of the study would see their picture and get their offers. Moreover, they were promised to be contacted and paid out if a future participant decided to accept their offers.

### Ultimatum Game Task

**Fig 1:**
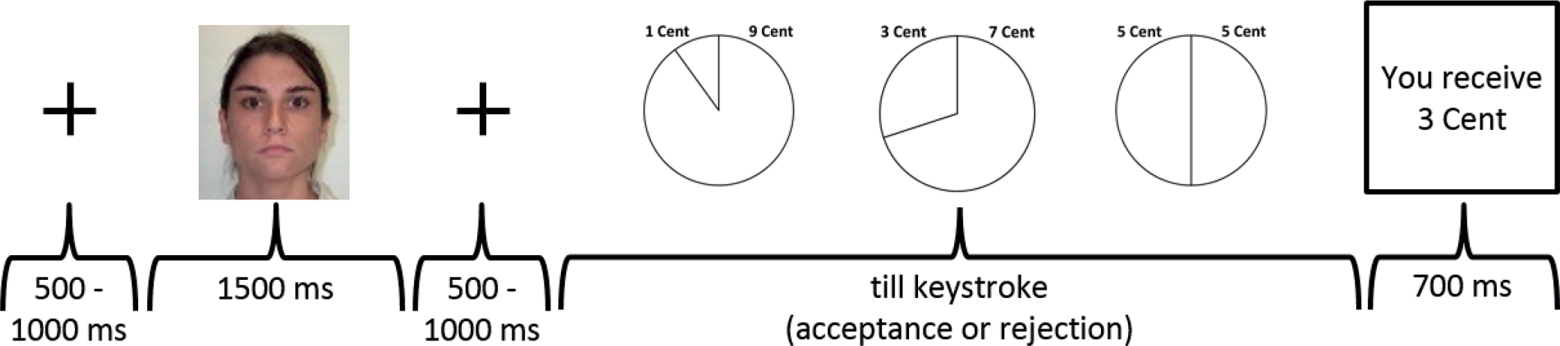
Sequence of an Ultimatum Game trial.

In the actual task, each participant played a series of 360 computerized Ultimatum Game trials, divided into four blocks of 90 trials each. Each trial started with a fixation cross appearing for 500 to 1000 ms (see Fig 1). After that, participants saw a randomized picture of one of 30 proposers (15 female, 15 male) with a neutral face (i.e., not showing any positive or negative emotions and not showing any teeth) [31] for 1500 ms. This was followed by another fixation cross for 500 to 1000 ms, and then the actual offer (1, 3, or 5 Cent out of 10 Cent) appeared as a pie chart. As soon as the participants accepted or rejected the offer by a keystroke, they were presented their earning on the present trial for 700 ms. Then, a new trial started.

Participants were told that the offers were made by real proposers and that both they and the proposers would be paid according to their decisions in the Ultimatum Game. In fact, each proposer made one very unfair (1 Cent), one slightly unfair (3 Cent) and one fair (5 Cent) offer in each block. The order of proposers and offers was randomized within each block.

As participants typically accept more unfair offers when knowing that they play against a computer instead of a human [19, 32], we chose to pay them more than their actual earnings. Specifically, the participants received 18 €, equaling the maximum possible earnings plus their promised extra fee of 6 €.

### Emotion Induction

At the beginning of each block, participants saw one out of four short movie clips for eliciting one of the target emotions *happiness*, *anger*, *fear*, and *neutral*. The order of the clips was randomized across participants. They were provided without audio and had a length between 64 and 236 seconds. The clips have been shown to successfully elicit their respective target emotions and to be comparable regarding content [33]. Clips were taken from the films *An Officer and a Gentleman* [34], *My Bodyguard* [35], *Halloween* [36], and *All the President’s Men* [33], for eliciting the emotions happiness, anger, fear, and neutral, respectively.

Participants were told that viewing the clips belongs to another experiment unrelated to the Ultimatum Game. In order to intensify the emotion induction, after seeing each film participants were asked to write down how, in their opinion, the protagonists felt in the scene that they just saw. Then, participants rated on seven-point-Likert scales how they felt themselves momentarily with regard to nine emotions, including the target emotions.

### Trait Affect Measures

After the experiment, participants were asked to fill out a German version of the PANAS [16] (German translation: [37]). This questionnaire consists of 20 emotions and moods, 10 for each of the dimensions *positive affect* and *negative affect*, and participants have to rate on five-point-Likert scales to which degree they sense each of the emotions. Positive affect “reflects the extent to which a person feels enthusiastic, active and alert” [16], whereas the negative affect dimension represents several aversive mood states, for example disgust, fear, and anger [16].

In our study, participants were asked to rate how they feel *in general*, thus ensuring that trait affect has been measured. As can be seen in Table 1, the internal consistency (Cronbach’s α) was good in our sample.

**Table 1:** Summary of the PANAS results.

	trait positive affect	trait negative affect
Minimum—Maximum	2.2–4.8	1.0–2.4
Mean (SD)	3.5 (0.57)	1.6 (0.49)
Cronbach’s α	.84	.87
Correlation	r = -.323 (p = .042, two-tailed)	

Each dimension consists of the mean score of 10 items, with each item rated on a Likert scale from 1 to 5.

### EEG Quantification

Participants were individually seated in an electrically shielded and dimly lit EEG cabin. For measurement, an EEG cap (Easycap GmbH, Herrsching/Breitbrunn, Germany) with 32 Ag/AgCl electrodes, collocated according to the 10/20 system, was applied. One additional electrode was placed under the left eye for recording a vertical electro-oculogram. Online reference was set to Cz, and impedances were kept below 5 kΩ. Signals were digitized using a BrainAmpDC amplifier (Brain Products, Gilching Germany) with a sampling rate of 250 Hz and a bandpass filter from 0.1 to 80 Hz.

Using the BrainVision Analyzer Software, Version 2.0.2.5859 (Brain Products GmbH, Gilching, Germany), a digital lowpass-filter (cut-off: 30 Hz) was applied for all electrodes, data were re-referenced to a common reference, and epoched from -200 to 1000 ms after presentation of the offer. Then, an Independent Component Analysis was applied for removing eye artefacts, and data were re-referenced to linked mastoids (TP9 and TP10). Former reference Cz was reinstated as an additional data channel. Finally, epochs were divided according to their experimental condition (offer amount x induced emotion), averaged for each participant and each electrode, and baseline-corrected from -200 to 0 ms before stimulus onset.

**Fig 2:**
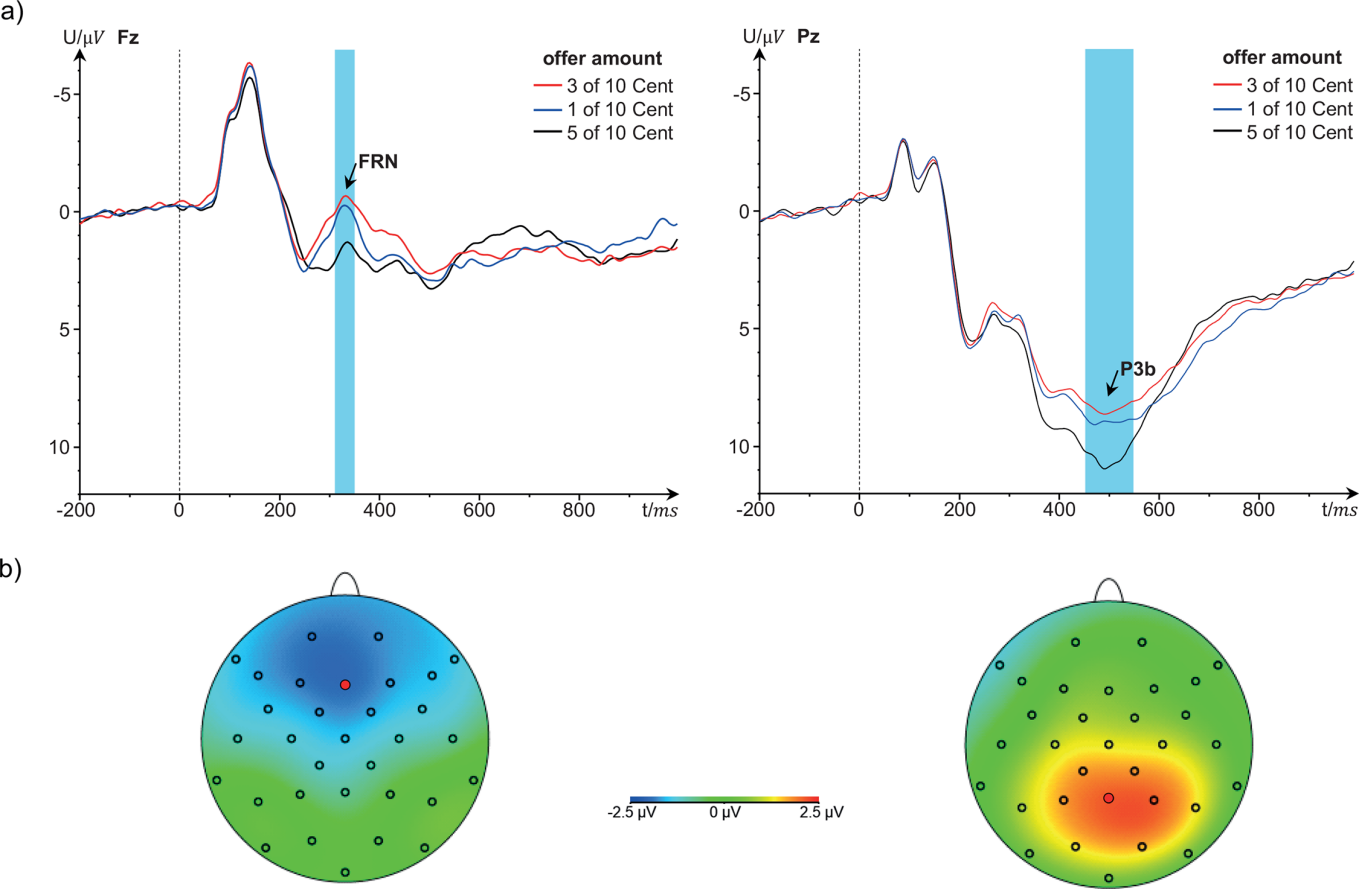
a) Event-related potentials following the presentation of the offer on electrode sites Fz (left) and Pz (right). b) Scalp distributions of the FRN (316 to 356 ms after presentation of the offer) and P3b (452 to 548 ms after presentation of the offer), evaluated as the difference waves between 3 Cent and 5 Cent, and 5 Cent and 3 Cent conditions, respectively.

The FRN was quantified as the average voltage between 316 and 356 ms after stimulus onset (presentation of the offer) on electrode site Fz, for each of the 12 experimental conditions. The P3b was defined as the average voltage between 452 and 548 ms after stimulus onset on Pz, for each experimental condition. The FRN shows a fronto-central distribution, whereas the P3b shows a parietal-central distribution (see Fig 2B), which is in line with previous studies [20, 29, 38].

### Statistical Analysis

For the emotion induction check, one-factor Analyses of Variance (ANOVAs) with induced emotion as independent variable, and self-reported ratings on each of the target emotions as dependent variable, were performed. In case of significant effects, contrast analyses were conducted.

For the evaluation of behavior, FRN, and P3b, multifactorial ANOVAs were performed for each dependent variable. We included offer amount (1, 3, or 5 out of 10 Cent) and induced emotion (happiness, anger, fear, and neutral) as within-factors, as well as trait positive affect and trait negative affect as covariates.

For behavior, we additionally calculated a panel data logistic regression with acceptance/rejection as binary dependent variable (not reported here). Results were qualitatively similar to the ANOVA.

In case of a significant main effect of one within-factor, or an interaction of the two within-factors, contrast analyses are reported. In case of a main effect of a covariate, the Product Moment-correlation of the covariate and the dependent variable was calculated. In case of an interaction between a covariate and a within-factor, interaction contrasts between the within-factor and the median-split of the covariate are depicted.

The significance level for all statistical tests was set to α = .05. For ANOVAs, partial eta-square-values (η_p_^2^) are reported as a measure of effect size. If a Mauchly-test indicated a violation of the assumption of sphericity, tests were Huynh-Feldt-corrected and marked with *HF*. In this case, non-adjusted degrees of freedom are reported.

## Results

### Emotion Induction Check

**Fig 3:**
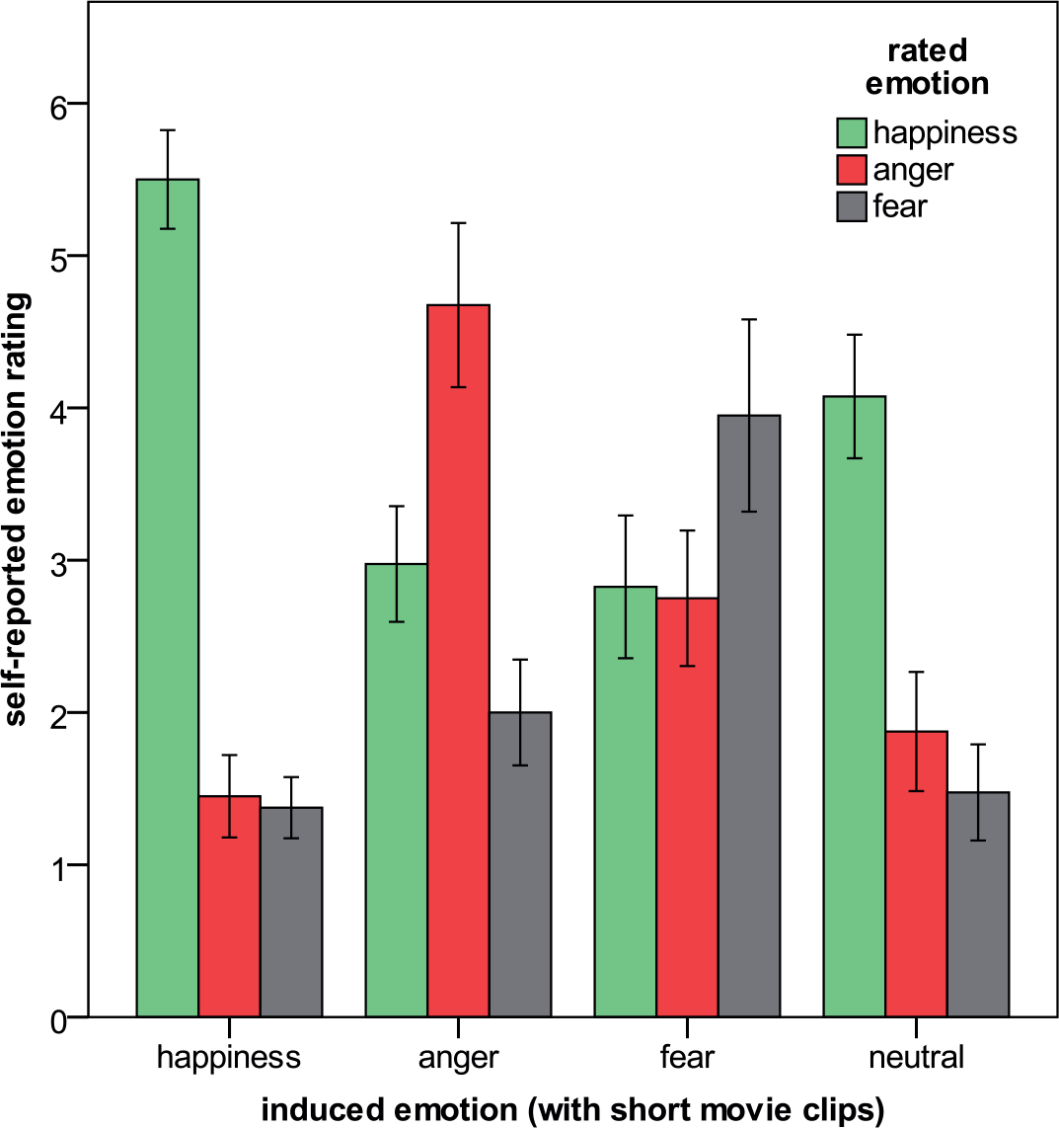
Self-reported emotion ratings as a function of induced emotion. Error bars represent 95% confidence intervals.

Analyses revealed significant main effects of self-reported happiness (F_3;117_ = 54.7; p < .001; η_p_^2^ = .584; HF), anger (F_3;117_ = 61.8; p < .001; η_p_^2^ = .613; HF), and fear (F_3;117_ = 46.1; p < .001; η_p_^2^ = .542; HF). As can be seen in Fig 3, participants reported more happiness (M = 5.50) after having seen the happy film compared to after having seen one of the other films (Ms were 2.98, 2.83, and 4.07 for the anger, fear, and neutral condition, respectively; all ps < .001, one-tailed). When participants were in the anger group, they rated themselves as being more angry (M = 4.67) than in the other conditions (Ms were 1.45, 2.75, and 1.87 for the happy, fear, and neutral condition, respectively; all ps < .001, one-tailed). After participants have seen the fear-inducing film, they reported more fear (M = 3.95) than in the other conditions (Ms were 1.38, 2.00, and 1.48 for the happy, anger, and neutral condition, respectively; all ps < .001, one-tailed).

### Behavior

**Fig 4:**
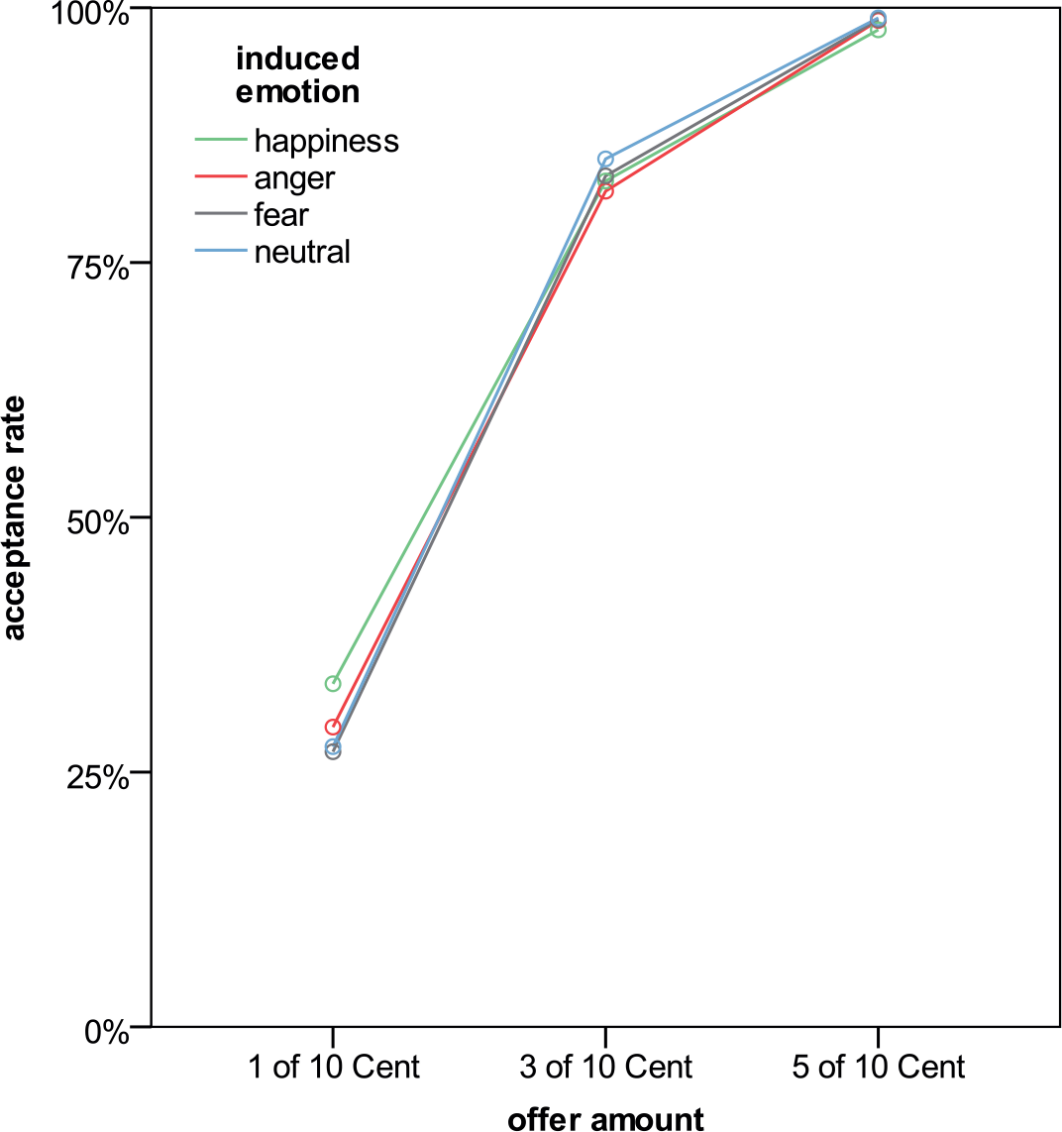
Acceptance rates as a function of offer amount and induced emotion.

A significant main effect of offer amount on acceptance rate was observed (F_2;74_ = 89.9; p < .001; η_p_^2^ = .708). More specifically, acceptance rates for fair (5 Cent) offers (M = 98.6%) were significantly higher than acceptance rates for slightly unfair (3 Cent) offers (M = 83.4%; p = .002, one-tailed) and very unfair (1 Cent) offers (M = 29.4%; p < .001, one-tailed). Also, acceptance rates for slightly unfair offers were significantly larger than those for very unfair offers (p < .001, one-tailed). Results are shown in Fig 4.

No significant main effect of induced emotion could be observed (F_3;111_ = 0.8; p = .487; η_p_^2^ = .022). However, the interaction *induced emotion x offer amount* reached significance (F_6;222_ = 2.5; p = .047; η_p_^2^ = .063; HF). When participants were in a happy mood, they accepted significantly more very unfair offers than when they were in the neutral condition (p = .034, one-tailed; Ms were 33.7% and 27.5% for the happy and neutral condition, respectively). However, acceptance rates for very unfair offers did not differ significantly between the neutral and the other two emotional conditions (ps were .437, two-tailed, and .421, one-tailed, for the comparisons to the anger and the fear condition, respectively; Ms were 27.0% and 29.4%, respectively). Also acceptance rates for slightly unfair offers did not differ relative to induced emotion (all ps>.070).

With regard to trait affect, neither a significant main effect of trait positive affect (F_1;37_ = 0.1; p = .796; η_p_^2^ = .002) nor a significant main effect of trait negative affect (F_1;37_ = 0.4; p = .513; η_p_^2^ = .012) could be observed. Also the interactions with offer amount, induced emotion, and *offer amount x induced emotion* did not reach significance (all ps>.065, partly HF).

### FRN

The scalp distribution of the FRN and event-related potentials on electrode site Fz are shown in Fig 2. A significant main effect of offer amount occurred (F_2;74_ = 13.6; p < .001; η_p_^2^ = .269; HF). More specifically, participants showed significantly larger FRN amplitudes (i.e., lower mean voltages) in response to very or slightly unfair offers (Ms were -0.079 μV and -0.549 μV, respectively), compared to fair offers (M = 1.468 μV; one-tailed ps were .001 and < .001, respectively). FRN amplitudes for very unfair and slightly unfair offers did not differ significantly (p = .130, two-tailed).

Again, no main effect of induced emotion was observed (F_3;111_ = 0.1; p = .966; η_p_^2^ = .002). Also the interaction *offer amount x induced emotion* did not reach significance (F_6;222_ = 0.98; p = .441; η_p_^2^ = .026).

**Fig 5:**
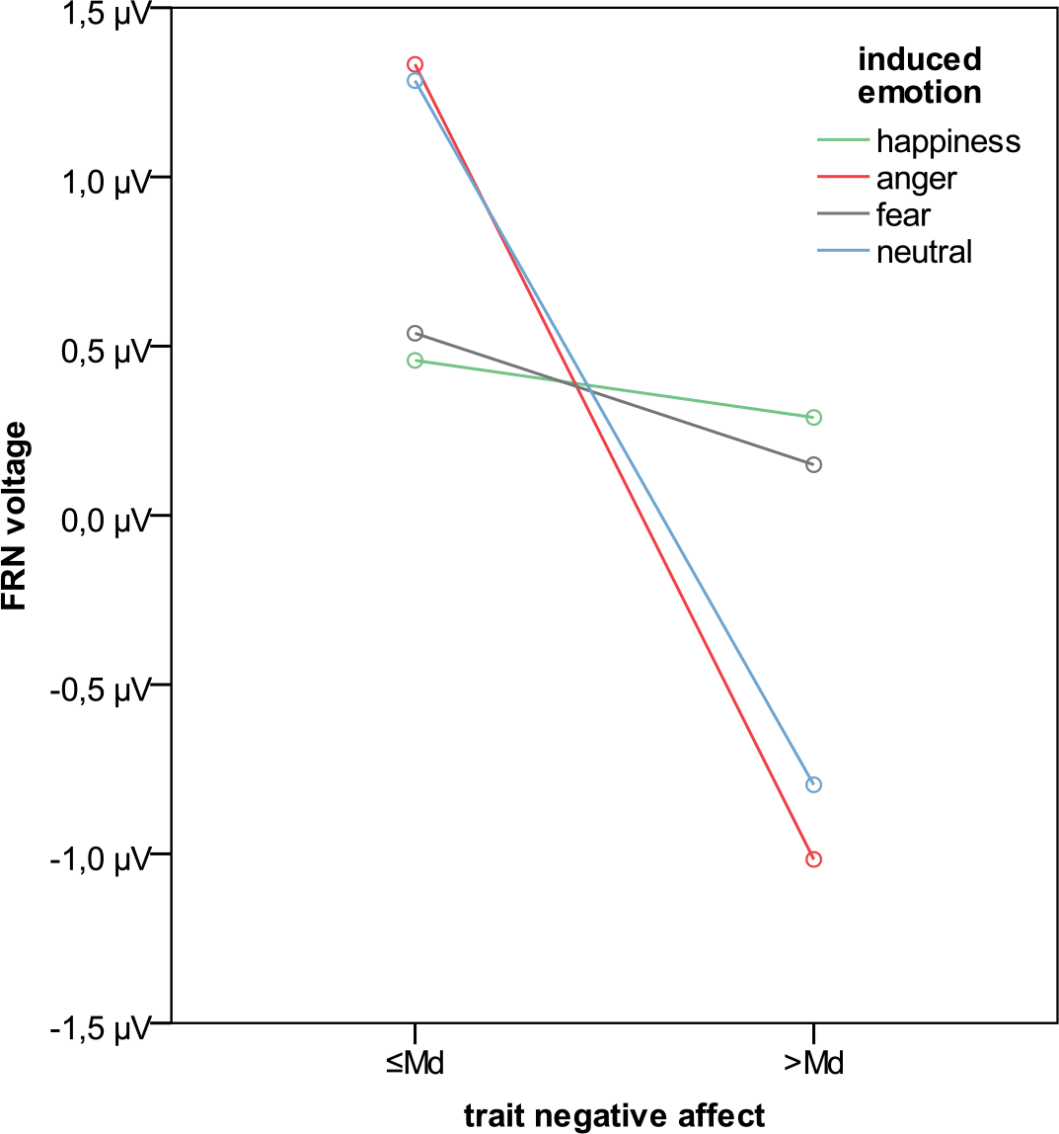
FRN voltages as a function of trait negative affect (median-split) and induced emotion.

Neither a main effect of trait positive affect (F_1;37_ = 2.0; p = .170; η_p_^2^ = .050) nor a main effect of trait negative affect (F_1;37_ = 0.2; p = .632; η_p_^2^ = .006) occurred. However, the interaction *induced emotion x trait negative affect* was significant (F_3;111_ = 3,6; p = .015; η_p_^2^ = .090). Interaction contrasts between induced emotion and the median-split of trait negative affect show that FRN amplitudes were significantly different between participants in the anger condition, compared to the happy condition (F_1;38_ = 7.0; p = .012, two-tailed) as well as for the anger, compared to the fear condition (F_1;38_ = 4.4; p = .042, two-tailed). As can be seen from Fig 5, in the anger condition, individuals with high levels of trait negative affect showed larger FRN amplitudes, compared to individuals low in trait negative affect (but not in the happy and fear condition). Interaction contrasts comparing the neutral condition with both happiness and fear failed to reach significance (neutral-happiness: F_1;38_ = 3.5; p = .071, two-tailed; neutral-fear: F_1;38_ = 3.3; p = .079, two-tailed). Additionally, interaction contrasts comparing anger and neutral, as well as happiness and fear were clearly non-significant (anger-neutral: F_1;38_ = 0.1; p = .717, two-tailed; happiness-fear: F_1;37_<0.1; p = .844, two-tailed). All other interactions of trait positive or negative affect to offer amount, induced emotion, and *offer amount x induced emotion* (i.e., all interactions except the interaction *induced emotion x trait negative affect* as described above) were not significant (all ps>.058, partly HF).

### P3b

The scalp distribution of the P3b and event-related potentials on electrode site Pz are shown in Fig 2. Again, a significant main effect of offer amount was found (F_2;74_ = 17.0; p < .001; η_p_^2^ = .315; HF). More specifically, participants showed significantly larger P3b amplitudes after having received fair offers (M = 10.554 μV) than after having received slightly unfair or very unfair offers (one-tailed ps were < .001 and .001, respectively; Ms were 8.437 μV and 8.960 μV, respectively). P3bs for slightly unfair and very unfair offers did not differ significantly (p = .076, two-tailed).

Both the main effect of induced emotion (F_3;111_ = 1.0; p = .379; η_p_^2^ = .027) and the interaction *offer amount x induced emotion* were not significant (F_6;222_ = 1.2; p = .316; η_p_^2^ = .031).

**Fig 6:**
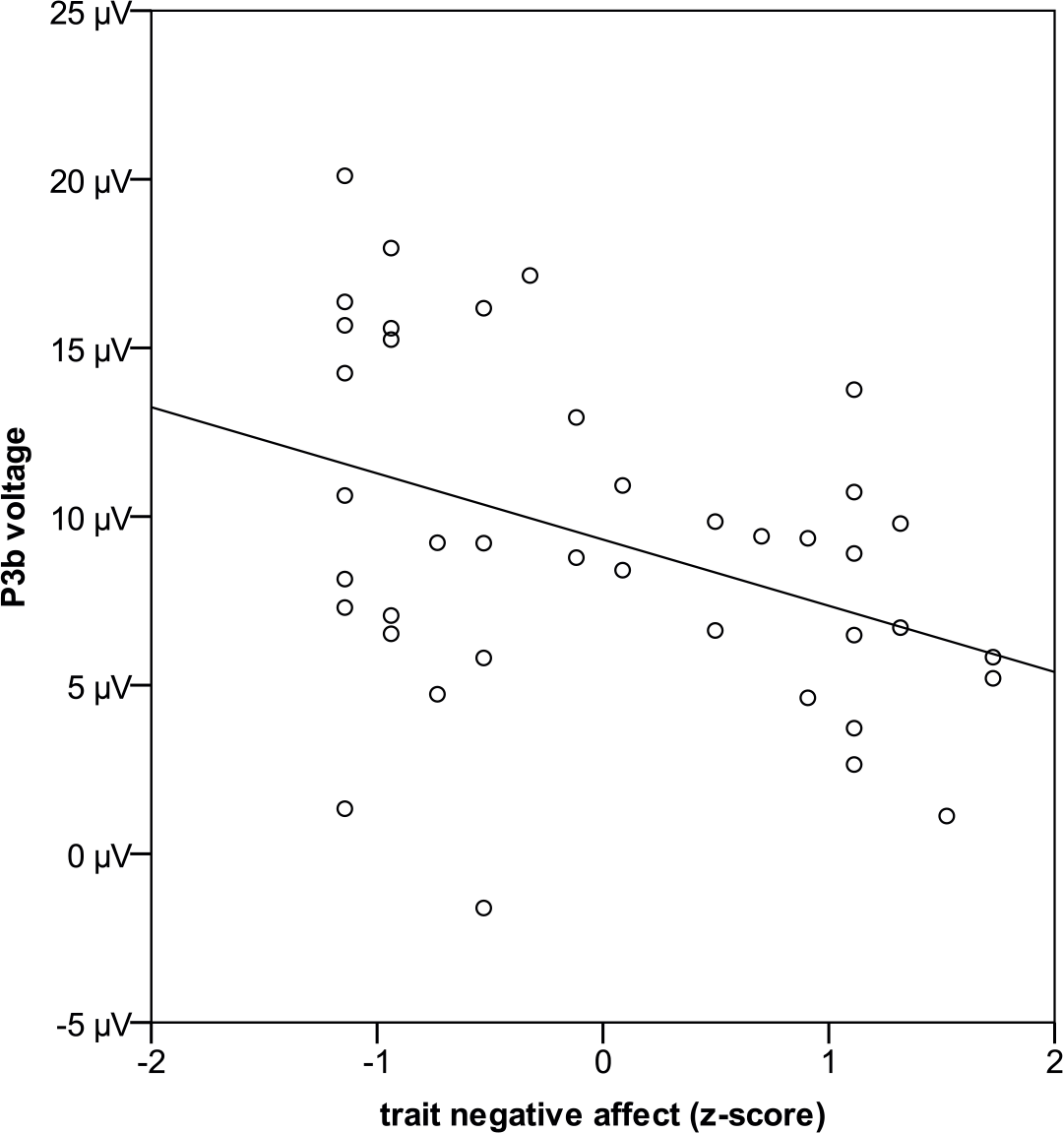
P3b amplitudes as a function of trait negative affect.

However, a main effect of trait negative affect occurred (F_1;37_ = 6.0; p = .019; η_p_^2^ = .139). Participants having low trait negative affect showed larger P3b amplitudes than those with high trait negative affect (r = -.392; see Fig 6). No main effect of trait positive affect (F_1;37_<0.1; p = .991; η_p_^2^ < .001) or any interaction of trait positive or trait negative affect to offer amount, induced emotion, or *offer amount x induced emotion* occurred (all ps>.147).

## Discussion

The present study investigated the influences of state and trait affect on social bargaining behavior and underlying neural correlates. We found an influence of state affect on decision making of participants acting as responders in the Ultimatum Game. Additionally, state and trait affect influenced and interacted with regard to neuropsychological measures, indicating that emotional states and traits play an important role in social behavior.

As in previous research [3] we found that the more unfair the offer was, the lower were the acceptance rates. This provides validity for our implementation of the experiment. Moreover, the emotion induction was successful as participants reported more happiness, fear, and anger after having seen the respective film, compared to after having seen each of the other films.

In line with our hypotheses, we found that participants accepted more very unfair offers if they were happy, compared to in a neutral mood. Although previous research had shown that unfair offers from smiling/happy looking proposers are more likely to be accepted than offers from non-smiling/neutrally looking proposers [12, 13], to our knowledge no previous study found that state happiness, induced prior to the Ultimatum Game task itself, leads to higher acceptance rates than neutral mood. In particular, Harlé and Sanfey [9] found lower acceptance rates to unfair offers when participants were in a sad, compared to a neutral mood, but they did not find significant differences between the happy and the neutral condition.

The observed differences were only present in very unfair (1 out of 10 Cent) offers, but not in slightly unfair (3 out of 10 Cent) offers. Moretti and di Pellegrino [15] reported effects of induced emotions for 1, 2, and 3 € offers, but not for 4, 5, and 6 € offers, out of a 10 € pot. Mussel et al. [12] found effects of differently valenced faces for 1, 2, 4, and 7 Cent offers, but not for 3, 5, and 6 Cent offers, out of a 12 Cent pot. It seems likely, thus, that the threshold until which state affect influences decisions in the Ultimatum Game lies at around 30% of the pot.

In contrast to our hypotheses, we did not find significant differences in behavior when comparing state anger and state fear to neutral mood. Unfortunately, we thus cannot draw conclusions on whether valence (positive vs. negative) or the motivational direction (approach vs. withdrawal) [14] of emotions are responsible for differences in acceptance behavior. Our finding that happy participants accepted significantly more unfair offers would have been predicted by both approaches [39, 40], as happiness is both a positive and an approach-motivated emotion.

Although Dunn et al. [7] reported higher acceptance rates to unfair offers for people low in trait positive affect and high in trait negative affect, we could not replicate this finding. This is the case even though we used the same questionnaire for measuring trait affect and we had a comparable sample. We think that this is an important finding (especially because we found influences of trait negative affect on event-related potentials) as it suggests that the influence of trait affect on behavior in the Ultimatum Game needs to be reconsidered in future research.

Regarding neurophysiology, we found that unfair offers elicited larger FRN amplitudes than fair offers. This is in line with previous research [13, 22–26] and may be explained through unfair offers representing negative feedback on how much the proposer is willing to offer to the responder, whereas fair offers represent positive feedback. The FRN had been shown to be larger for negative, compared to positive feedback [20]. Additionally, we found no significant difference between slightly unfair and very unfair offers, indicating that the FRN reflects a binary categorization of the outcome as either good or not good. These results are in line with previous research on gambling and reinforcement sensitivity tasks with three outcomes (e.g., good, bad, and neutral), which found larger FRN for bad and neutral, compared to good outcomes, but no difference between neutral and bad outcomes [39, 40], and with studies which failed to reveal an influence of outcome magnitude on FRN amplitude [28, 39, 41].

To our knowledge, this is the first study that investigated the influences of emotions in the Ultimatum Game and simultaneously recorded EEG data. We found a significant interaction between state affect and trait negative affect. The interpretation of contrasts revealed that when participants were in an angry mood, trait negative affect predicted FRN amplitudes with larger FRN amplitudes for individuals with high, compared to low levels on trait negative affect. However, this effect seemed to be absent when participants were happy or experienced fear. The effect of trait negative affect on FRN amplitude in the neutral condition was, descriptively, similar to the effect observed in the angry condition, but did not differ significantly from fear and happiness (.07<ps < .08).

As trait negative affect indicates subjective distress and various aversive mood states [16], trait negative affect is positively associated with rumination [42]. The higher tendency of people high in trait negative affect to ruminate may be reflected in larger FRN amplitudes. In line with this assumption, Moser, Moran, Schroder, Donnellan, and Yeung [43] report meta-analytic results on the prediction of FRN amplitudes and anxious apprehension, with larger FRN amplitudes for individuals high, compared to low in anxious apprehension. The authors theorize that the larger FRN amplitudes might reflect enhanced transient “reactive” control to distracting rumination and worries which interfere with the ability of anxious individuals to focus on affectively neutral tasks. This is at least the case in conditions when participants ruminate, which is, when they are in a neutral mood or are angry. However, when people are happy, they might not ruminate because happy people value their current situation, thus not showing anxious apprehension. Consequently, their general tendency to exhibit increased levels of rumination might be overwritten in this condition, resulting in equal FRN amplitudes between people high and low in trait negative affect.

As fear and anxiety are related concepts, it might at first glance seem surprising that the effect of trait negative affect on FRN amplitudes was not apparent in the fear condition. However, an important differentiation has been made between anxious apprehension and anxious arousal [43–45]. The latter is defined by physiological arousal elicited by clear and present threats, and readily corresponds to our fear condition in which participants had to watch a thrilling horror movie clip. Indeed, people experiencing fear tend to flee without thinking too much. Contrary, anxious apprehension is characterized by rumination and worry elicited by ambiguous future threats. Therefore, as our fear condition lacks the characteristics of rumination and worry, the absence of an effect of trait negative effect on FRN amplitudes in this condition is actually in line with our interpretation of rumination as a potential explaining mechanism. Comparing states of anxious apprehension and anxious arousal directly regarding behavior in the Ultimatum game points towards an interesting area for future studies.

Apart from FRN, we investigated the P3b component. Similar to Ma et al. [26] we found that P3b amplitudes were larger after fair, compared to unfair offers. Nieuwenhuis et al. [29] suggest that the locus coeruleus–norepinephrine system is involved in evaluating stimuli and in deciding whether or not to respond to them, processes which are dependent on the motivation to respond to the stimuli. They also found evidence that the outcome of these processes elicit the P3b component. In line with this proposal, the P3b component is larger when a stimulus is a target [46]. The increased P3b component after fair offers in our study might thus represent increased pleasure in response to the stimuli and increased motivation to engage in future activities (e.g., playing more Ultimatum Game trials; see also [30, 47]).

Importantly, we also found a main effect of trait negative affect on P3b amplitudes, with people high in trait negative affect having lower P3b amplitudes than those low in trait negative affect. Thus, people that regularly experience distress and aversive mood states might experience less pleasure and less motivation to engage in future activities, resulting in lower P3b amplitudes. Substantially, this effect is true for both fair and unfair offers, as no interaction of trait negative affect with offer amount occurred. Given that trait negative affect is linked to depression severity [7, 18], our results are also in line with reports of an inverse relation between P3 amplitudes and depressive symptoms [48, 49].

A potential limitation of our study is that we cannot distinguish between the effects of offer fairness and those of the different monetary value of the offers because higher fairness automatically means higher monetary value. Others have used different pot sizes (e.g., $2 out of a $10 pot is unfair, but $2 out of a $4 pot is fair) to overcome this issue [30, 47, 50]. However, effects have been shown to be relatively stable across different pot sizes and to occur mainly due to varying fairness [3, 30, 47, 50]. Therefore, we believe that this is just a minor limitation for our study.

Apart from that, future studies would benefit from investigating the different motives that might drive behavior in the Ultimatum Game. Whereas the rejection of unfair offers might be an altruistic act of punishing unfair proposers in order to make them behave more reciprocally [8], new research indicates that at least a part of the responders act spitefully, that is that they try to reduce the payoff of others intentionally while trying to not being subjugated by the proposer [51–53]. On the other hand, accepting unfair offers is not always a way to maximize one’s own payoff, but can also be motivated by generosity as some participants even accept offers that pay them nothing and give all of the pot to the proposer [54, 55].

In sum, we were able to show that happiness leads to increased acceptance rates of very unfair Ultimatum Game offers. Also, we showed that emotions influence neurophysiological measures in this paradigm, with an interaction of state and trait negative affect influencing the FRN, and high trait negative affect leading to decreased P3b amplitudes. Future studies may further address this issue by replicating our results and by investigating whether the revealed biases are also present in clinical samples.

## Supporting Information

S1 Dataset Dataset (SPSS File). (SAV)
